# Characterization of Key Aroma Compounds in Aged Chinese *Nongxiangxing* Baijiu Based on Sensory and Quantitative Analysis: Emphasis on the Contribution of Trace Compounds

**DOI:** 10.3390/molecules30142963

**Published:** 2025-07-14

**Authors:** Peiqi Li, Yuting Ling, Xiaomei Shen, Chengcheng Liang, Youhong Tang, Shan Chen, Lisa Zhou Wang, Shuang Chen, Anjun Li, Yan Xu

**Affiliations:** 1 Laboratory of Brewing Microbiology and Applied Enzymology, Key Laboratory of Industrial Biotechnology of Ministry of Education, School of Biotechnology, Jiangnan University, 1800 Lihu Avenue, Wuxi 214122, China; 18101536750@163.com (P.L.);; 2 Anhui Province Key Laboratory of Intelligent Solid-State Fermentation Technology, Bozhou 236820, China

**Keywords:** *Nongxiangxing* Baijiu, aging process, GC-O/MS, aroma recombination, aroma omission

## Abstract

The characteristics and complexity of Baijiu are inseparable from the promotion of aging. While the impact of compounds such as alcohols, esters, and acids on the aroma of aged Baijiu has been extensively studied, the role of other trace compounds in the aging process should not be overlooked. To further investigate the relationship between volatile compounds and the aging of *Nongxiangxing* Baijiu, sensomics research methods were employed to analyze profiles of young and aged *Nongxiangxing* Baijiu. In this study, a total of 94 aroma compounds were analyzed in both young and aged *Nongxiangxing* Baijiu by GC-O/MS. Among these, 12 aroma compounds significantly associated with the aging process were identified by quantification and odor activity values (OAVs). Furthermore, the omission tests result showed that 4-methyl-2-methoxyphenol (2066.79 μg/L), benzaldehyde (3860.30 μg/L), β-phenylethanol (5638.85 μg/L), 3-(methylsulfanyl)propan-1-ol (8.82 μg/L), 3-(methylsulfanyl)propanal (15.91 μg/L), and linalool (17.36 μg/L) were key aroma compounds of aged *Nongxiangxing* Baijiu. This study reveals that trace compounds contribute to the distinct aroma complexity of aged *Nongxiangxing* Baijiu, providing a foundation to support aging process analysis.

## 1. Introduction

Baijiu, as a flavor product, has its aroma characteristics playing a crucial role in consumers’ selection process. Baijiu is a distilled liquor primarily produced from cereals and grains. It employs various fermentation starters—including *daqu*, *xiaoqu*, *fuqu*, enzyme preparations, and yeast—to facilitate starch saccharification and fermentation. The production process encompasses sequential stages of steaming, saccharification, fermentation, distillation, aging, and blending [1]. The aging process of Baijiu is a vital step in its production; indeed, the enhancement of aroma is significantly noticeable after aging, leading to a marked improvement in quality [2]. Industry surveys (e.g., the *2025 China Baijiu Market Mid-Term Research Report) indicate significantly higher consumer acceptance and consumption volume for extensively aged Baijiu [3]. Investigating the science behind Baijiu aging therefore addresses critical market demands while advancing modernization of traditional distilling through evidence-based research. Consequently, there is a growing interest in studying the aging process. By elucidating the changes that occurred during aging and the aroma benefits of properly stored Baijiu, we can better guide flavor regulation throughout the storage process, ultimately enhancing the quality of traditional Chinese Baijiu. *Nongxiangxing* Baijiu is one of the most significant types in the Baijiu category. In 2021, its sales revenue constituted the dominant share of China’s Baijiu market, establishing itself as the important revenue source within the industry [4]. *Gujinggong*, a distinctive variety produced in the *Huanghuai* production area, is a brand of unique and traditional *Nongxiangxing* Baijiu. While existing studies have characterized its key aroma-active compounds, systematic investigations into aging-related critical substances remain largely unexplored [5]. Elucidating the scientific principles of maturation requires identifying key aromatic compounds exhibiting significant concentration variations during storage periods. To further reveal the underlying reasons for consumers’ preference toward well-aged Baijiu products with superior flavor quality is important.

Research into the flavor chemistry of *Nongxiangxing* Baijiu has a long-standing history. Li was the first to identify the contents of ethyl hexanoate and ethyl lactate, which are the main aroma compounds in concentrated Baijiu [6]. Fan et al. analyzed *Nongxiangxing* Baijiu using HS-SPME in combination with GC-O-AEDA. Their findings revealed that esters, particularly ethyl hexanoate, were the predominant contributors to the aroma of Baijiu samples [7]. Gong identified 50 aroma substances in commercial *Nongxiangxing* Baijiu through sensory analysis and briefly examined the differences in compounds among various Baijiu samples [8].

In aging studies involving two other major aroma types of Baijiu, Wang et al. concluded that terpenoids, aldehydes, ketones, and sulfur compounds are key contributors to the aroma of aged Baijiu after analyzing samples of *Qingxiangxing* Baijiu [9]. Similarly, they found that furanones, aldehydes, and pyrazines are associated with the aging aroma in *Jiangxiangxing* Baijiu [10]. For *Nongxiangxing* Baijiu research, Niu et al. undertook a comprehensive study of *Nongxiangxing* Baijiu, employing Pearson’s correlation coefficient to uncover characteristic compounds linked to sensory attributes. They identified eight esters and acids associated with the aging aroma [11]. Current research on *Nongxiangxing* Baijiu has predominantly focused on alcohols, esters, and acids, with limited attention paid to other trace substances. Consequently, it was hypothesized that non-alcohol/ester/acid compounds play a pivotal role in shaping the maturation-driven aroma profile of *Nongxiangxing* Baijiu during aging. This hypothesis was supported by preliminary evidence indicating that trace compounds such as pyrazines, sulfur-containing molecules, and phenolic derivatives exhibit significant concentration shifts correlated with storage duration. However, systematic validation of their sensory contributions through sensory-oriented quantitative analyses, particularly across varying aging periods, remains underexplored.

In this study, we selected crude *Nongxiangxing* Baijiu samples with varying years of aging and employed molecular sensory science to conduct both qualitative and quantitative analyses of the Baijiu’s aroma with a focus on sensory orientation. We reconstructed and identified the key aroma compounds through recombination and omission experiments. This research aims to provide a foundation for understanding the aging process of *Nongxiangxing* Baijiu, ultimately contributing to a deeper analysis of its aging mechanisms and offering a scientific basis for optimizing the aging process.

## 2. Results and Discussion

### 2.1. Aroma Profile of Different Aging Years of Baijiu Samples

**Figure 1:**
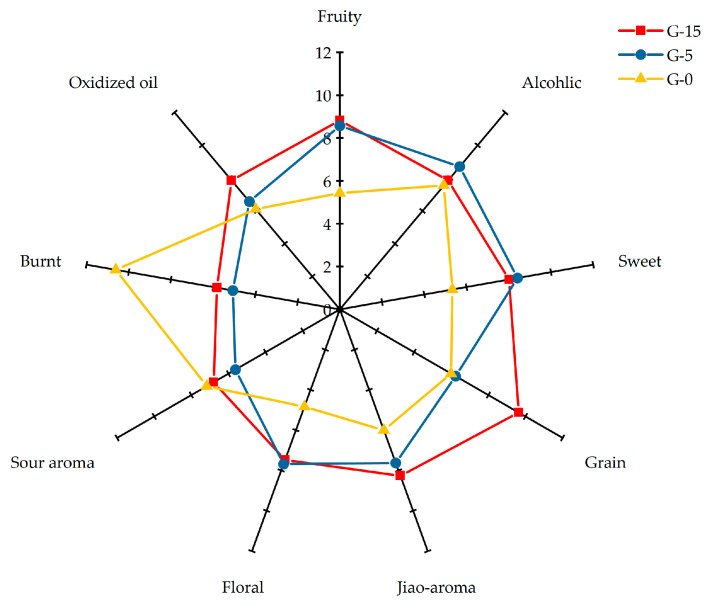
Sensory profiles of Baijiu with different aging times.

A visual investigation of the aroma profile characteristics of Baijiu samples (G-15, G-5, G-0) was conducted using nine recognized odor descriptors (Figure 1). Overall, the aged sample (G-15) exhibited significantly lower intensities in burnt and sour aromas compared to the younger sample (G-0), while higher intensities were observed in the remaining seven sensory attributes. Notably, G-15 demonstrated statistically elevated intensities in fruity, sweet, jiao-aroma (cellar-derived aroma), and floral notes relative to G-0, accompanied by a marked reduction in burnt intensity. To elucidate the underlying causes of aroma divergence among samples with varying storage durations, a comparative analysis of flavor compounds was performed using comprehensive Aroma Dilution Analysis combined with Gas Chromatography-Olfactometry (cADEA-GC-O).

### 2.2. Identification of Aroma Compounds in Young and Aged Nongxiangxing Baijiu by GC-O/MS

The odorants present in Baijiu samples are low in concentration yet highly complex. Liquid–liquid extraction (LLE) effectively isolates and concentrates these aroma compounds. The concentrated extract was further analyzed using cAEDA to determine the flavor dilution factor (FD) value of each detectable odorant via gas chromatography-olfactometry (GC-O). This complexity will be investigated further through a detailed analysis of the individual compounds involved. As shown in Table 1, a total of 94 distinct aroma compounds were identified in the Baijiu samples, which include 27 types of esters, 16 types of alcohols, 11 types of acids, 13 types of aldehydes and ketones, 4 types of terpenoids, 3 types of lactones, 5 types of aromatics, 4 types of furans, 3 types of sulfur-containing compounds, 2 types of pyrazines, and 6 types of phenolic compounds. Esters are important to Baijiu, and they typically impart a floral, fruity, and sweet character to Baijiu [12]. The flavor attributes of alcohol compounds were observed to vary significantly depending on their functional group configurations. Saturated alcohols were predominantly associated with alcoholic notes, while unsaturated alcohols contributed to floral, fruity, and mushroom-like aromas. Complex compounds containing hydroxyl groups were found to impart multifaceted sensory characteristics, such as honey, almond, grassy, and rose nuances [13]. Acids play a vital role in the aromatic presentation of alcoholic beverages, often introducing a sour note. However, certain substances, like butanoic acid, can present a distinctly sour aroma.

### 2.3. Quantification of Aroma Compounds in Baijiu Samples and Trace Compounds OAV Analysis

Trace compounds were defined as those with concentrations typically below 0.01% of the total sample mass (i.e., <100 ppm). While the contributions of higher-concentration compounds to the overall flavor profile of *Nongxiangxing* Baijiu had been extensively characterized, the effects of most trace compounds on odor characteristics were found to differ markedly, often exerting greater influence on the sensory properties of alcoholic products compared to their higher-concentration counterparts [14]. To systematically evaluate the role of trace aroma compounds, the concept of Odor Activity Value (OAV) was introduced. It is defined as the ratio of the concentration of a specific odorant in a sample to its odor detection threshold concentration in an appropriate matrix, which quantifies their contributions to the holistic sensory experience based on both concentration and odor detection thresholds.

A total of 85 compounds were accurately quantified, with their respective calibration curves, recovery rates, and linearity ranges summarized in Supplementary Table S1. Among them, 63 compounds were subsequently screened for Odor Activity Value (OAV) calculations. Comparative ratios of OAV data between G-15 (aged) and G-0 (young) samples were computed to assess the extent of changes in trace compounds during storage maturation and to determine their contributions to the aroma profiles of both young and aged Baijiu.

As presented in Table 2, absolute quantitative concentrations of trace compounds exhibited minimal changes during Baijiu aging. However, when assessing odor contribution through Odor Activity Value (OAV) calculations incorporating detection thresholds, the impact of aging became pronounced. For instance, while the concentration of β-damascenone increased by 3.95-fold after 15 years of aging, its absolute concentration change was merely 10 μg/L. This demonstrates that sensory significance cannot be inferred solely from absolute concentration variations. These findings demonstrated that the use of OAV ratios provided a more objective and rational approach to assess the impact of trace compound variations on the overall flavor profiles of aged and young Baijiu, as this metric inherently integrates both quantitative changes and aroma olfactory sensitivity.

Eighteen compounds were identified with Odor Activity Value (OAV) ratios exceeding 2 between aged (G-15) and young (G-0) Baijiu, indicating at least a twofold increase in their concentrations. Conversely, nine compounds exhibited a reduction in concentrations by at least half. These aroma compounds, characterized by pronounced disparities in OAV values, were hypothesized to contribute significantly to the divergent aroma profiles between aged and young Baijiu samples.

### 2.4. Variation Pattern of Trace Aroma Compound Concentration During Aging of Nongxiangxing Baijiu

**Figure 2:**
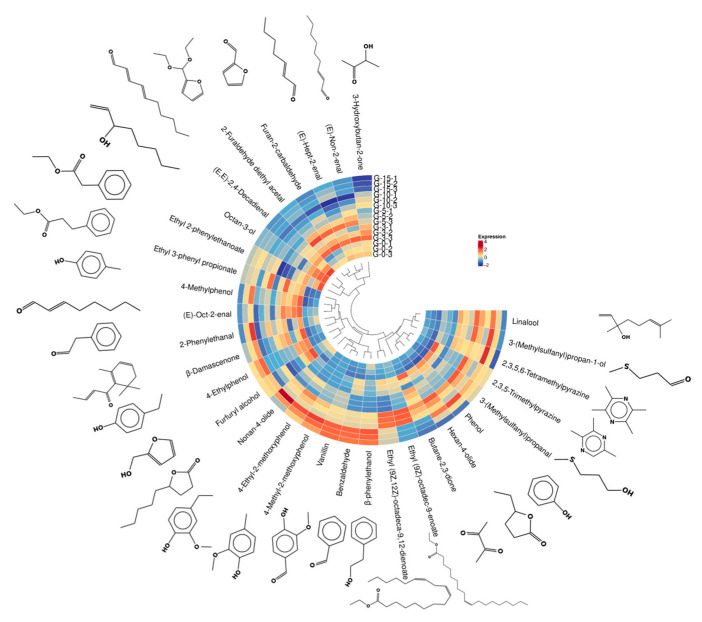
Concentration distribution of 31 trace aroma compounds in 15 *Nongxiangxing* Baijiu samples aged for different years.

The Baijiu samples aged 0, 5, and 15 years analyzed in this study were produced by the same manufacturer, with no alterations in core production processes or raw materials during this period. However, variations in and production instability may have partially influenced the observed discrepancies in final flavor quality. Variations in climatic conditions significantly influence the microbial community structure and flavor substance content within cellar soil [22]. Therefore, to minimize the confounding effects of climatic variability and production instability, samples produced from distinct fermentation pits representing different batches within the same vintage year were selected as parallel replicates. These samples underwent prolonged storage under identical conditions, facilitating focused analysis and discussion pertaining to the aging process. To further investigate the evolution of trace compounds—particularly those beyond common alcohols, acids, and esters—during storage, a total of 15 *Nongxiangxing* Baijiu samples aged 0, 3, 5, 10, and 15 years were systematically evaluated. The concentration differences of 31 compounds across these samples were visualized using heatmaps, revealing distinct clustering patterns correlated with aging duration (Figure 2).

**Figure 3:**
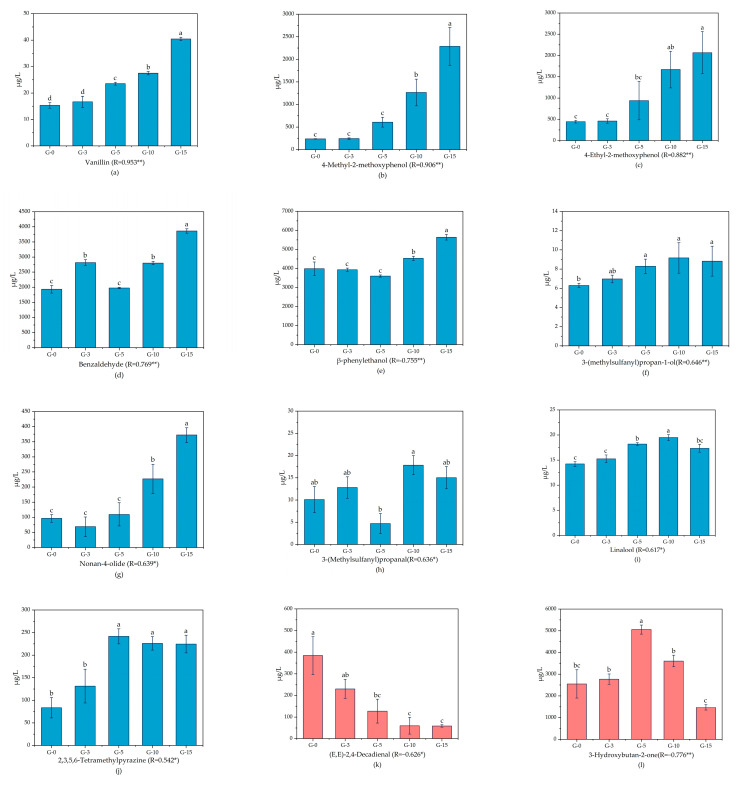

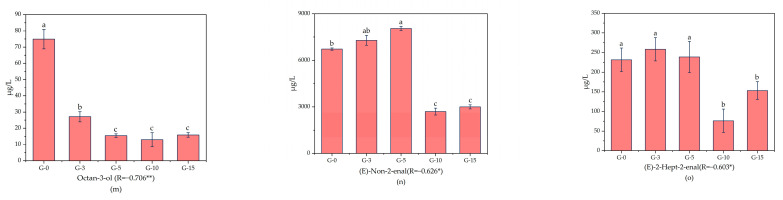
Average concentrations of trace aroma compounds Vanillin (**a**), 4-Methyl-2-methoxyphenol (**b**), 4-Ethyl-2-methoxyphenol (**c**), Benzaldehyde (**d**), β-phenylethanol (**e**), 3-(methylsulfanyl)propan-1-ol (**f**), Nonan-4-olide (**g**), 3-(Methylsulfanyl)propanal (**h**), Linalool (**i**), 2,3,5,6-Tetramethylpyrazine (**j**), (*E*,*E*)-2,4-Decadienal (**k**), 3-Hydroxybutan-2-one (**l**), Octan-3-ol (**m**), (*E*)-Non-2-enal (**n**), (*E*)-2-Hept-2-enal (**o**) in 0, 3, 5, 10, and 15 year old *Nongxiangxing* Baijiu samples and their correlation with aging time. Bars labeled with differing superscript letters (a, b, c, ab, bc) denote significant differences (*p* < 0.05) based on ANOVA with Duncan’s multiple range test. Shared letters indicate non-significant pairwise comparisons. * denotes significant correlation with time (*p* < 0.05); ** denotes stronger correlation (*p* < 0.01).

Statistical analysis revealed that the concentration changes of 15 out of 31 trace compounds were significantly correlated with aging duration. Among these, ten compounds—vanillin, 4-methyl-2-methoxyphenol, 4-ethyl-2-methoxyphenol, benzaldehyde, β-phenylethanol, 3-(methylsulfanyl)propan-1-ol, Nonan-4-olide, 3-(Methylsulfanyl)propanal, linalool, and 2,3,5,6-tetramethylpyrazine—exhibited a positive correlation with increasing storage time. In contrast, five compounds, (*E*,*E*)-2,4-decadienal, 3-hydroxybutan-2-one, octan-3-ol, (*E*)-non-2-enal, and (*E*)-2-hept-2-enal, displayed a negative correlation with prolonged aging (Figure 3).

Among the compounds analyzed, vanillin exhibited the strongest correlation with aging duration, a trend consistent with prior studies in wine [23], beer [24], and rice wine [25], where vanillin concentrations were reported to increase over time. The current findings align with this established pattern. However, the concentration of 4-vinylguaiacol remained below the detection limit. This observation suggests that while 4-vinylguaiacol may undergo conversion to vanillin, the resultant vanillin levels (significantly below its odor threshold of 438.52 μg/L) were insufficient to offset the depletion of 4-vinylguaiacol, leading to its progressive reduction during aging. According to Nele et al. [24], 4-vinylguaiacol undergoes oxidative conversion to vanillin as its primary metabolic pathway. This transformation is modulated by environmental factors, including oxygen availability and pH. Their findings demonstrated maximal vanillin yield at pH 10. However, in *Nongxiangxing* baijiu (typical pH 3.0–5.5), oxygen concentration likely constitutes the dominant factor governing the conversion efficiency of 4-vinylguaiacol to vanillin.

### 2.5. Aroma Recombination and Omission

**Figure 4:**
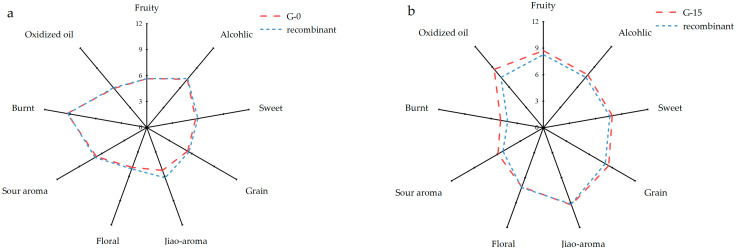
Aroma profiles of G-0 Baijiu (**a**), G-15 Baijiu (**b**), and their aroma-reconstituted models.

To investigate key aroma-active compounds, a common approach involves systematic omission tests coupled with sensory evaluation. This assesses whether the omission of an individual compound results in a significant divergence from the overall aroma profile. Consequently, reconstitution experiments are essential. These experiments require the preparation of a sensorially accurate replica of the original sample’s aroma profile. This is achieved by quantitatively recombining the maximum feasible number of identified volatile compounds, utilizing authentic chemical standards at their accurately quantified concentrations. The validated reconstitution model then serves as the baseline for subsequent omission studies. Aroma recombination tests were performed by reconstituting volatile compounds with odor activity values (OAVs) > 0.1 into a 60% (*v*/*v*) ethanol–water solution, based on the precise quantitative results of G-0 (young Baijiu) and G-15 (aged Baijiu) (Figure 4). The reconstituted samples were then subjected to comparative analysis of aroma characteristics against the original liquors. This approach enabled the establishment of a predictive model to delineate the distinct aroma profiles of young and aged *Nongxiangxing* Baijiu.

The aroma profile plots revealed no significant differences in flavor intensity between the reconstituted models and the original samples across nine sensory attributes: fruity, jiao-aroma, sweet, grain, alcoholic, burnt, floral, sour aroma, and oxidized oil. However, discrepancies were observed in specific attributes. For the G-15 model, the intensities of burnt, oxidized oil, and fruity notes were not fully replicated compared to the original aged samples. Similarly, the G-0 model failed to completely restore the jiao-aroma characteristic of the young Baijiu. Despite these limitations, the reconstituted Baijiu samples were demonstrated to effectively reconstruct the overall flavor profile of the originals when evaluated against the nine aroma descriptors. These findings highlighted the critical role of the identified aroma compounds in defining the sensory identity of Baijiu and validated the precision of the quantitative analytical methodology employed.

In the reconstituted aged Baijiu samples, the contribution of ten key aroma compounds—vanillin, 4-methyl-2-methoxyphenol, 4-ethyl-2-methoxyphenol, benzaldehyde, β-phenylethanol, 3-(methylsulfanyl)propan-1-ol, Nonan-4-olide, 3-(methylsulfanyl)propanal, linalool, and 2,3,5,6-tetramethylpyrazine—to the aroma profile of aged *Nongxiangxing* Baijiu was systematically investigated using the triangular test method. These compounds had been previously identified as exhibiting significant positive correlations with storage duration.

As shown in Table 3, highly significant differences (*p* < 0.001) were detected by 18, 16, and 18 out of 20 panelists for the omission models of 4-methyl-2-methoxyphenol, 3-(methylsulfanyl)propan-1-ol, and 3-(methylsulfanyl)propanal, respectively. These results indicated that the smoky odor attributed to 4-methyl-2-methoxyphenol, the roasted meat-like aroma from 3-(methylsulfanyl)propan-1-ol, and the musty tomato-like note associated with 3-(methylsulfanyl)propanal are critical trace aroma compounds contributing to the aged *Nongxiangxing* Baijiu profile. Significant differences (*p* < 0.01) were also observed when β-phenylethanol, benzaldehyde, and linalool were omitted from the reconstituted samples, underscoring their roles in imparting floral and sweet notes, which are essential to the characteristic flavor of aged *Nongxiangxing* Baijiu. In contrast, the omission of vanillin, 4-ethyl-2-methoxyphenol, nonan-4-olide, and 2,3,5,6-tetramethylpyrazine did not yield statistically significant differences. Notably, although vanillin exhibited a strong correlation with aging duration, its concentration remained far below the odor detection threshold, rendering it imperceptible. Based on these findings, six compounds—4-methyl-2-methoxyphenol, 3-(methylsulfanyl)propan-1-ol, 3-(methylsulfanyl)propanal, β-phenylethanol, benzaldehyde, and linalool—were identified as key trace aroma components defining the maturation-driven sensory characteristics of *Nongxiangxing* Baijiu.

Phenolic compounds are primarily responsible for imparting woody and smoky notes in alcoholic beverages. Previous studies have suggested that their formation may be derived from the thermal degradation of lignin or the aromatization of aliphatic compounds under high-temperature conditions [26,27]. In aged Gujinggong Baijiu, two phenolic derivatives—4-methyl-2-methoxyphenol and 4-ethyl-2-methoxyphenol—were identified as trace aroma compounds exhibiting significant correlations with aging duration. Notably, 4-methyl-2-methoxy2phenol, while unreported in alcoholic beverages, has been widely documented as a key aroma contributor in smoked foods (e.g., ham) due to its smoky nuances [28]. Conversely, 4-ethyl-2-methoxyphenol is recognized as a metabolic by-product of *Brettanomyces yeast* in wine, enhancing complexity at moderate levels but imparting barnyard-like odors at elevated concentrations [29]. In the context of *Nongxiangxing* Baijiu, these phenolic compounds may shift sensory perceptions in aged samples: panelists predominantly associated them with grain-like nuances rather than the undesirable burnt notes characteristic of younger samples. This divergence underscores their role in refining the aroma profile during maturation.

The concentrations of benzaldehyde and β-phenylethanol, two aromatic compounds, were observed to increase significantly with extended aging duration, exhibiting almond-like and rose-like aromas, respectively. This elevation in their levels was found to further enhance the maturation characteristics of Baijiu, potentially amplifying the sweet and floral flavor profile of aged samples. Notably, the role of aromatic compounds in oxidative reactions has been documented in wine studies; for instance, Hernández-Orte demonstrated that such compounds are closely associated with oxidation-driven transformations during wine aging [30], a mechanism that may also underpin their dynamics in Baijiu systems.

Sulfur-containing compounds have been recognized as critical aroma contributors in numerous Baijiu varieties due to their exceptionally low odor thresholds, which enable them to impart intense characteristic aromas even at trace levels. Among these, 3-(methylsulfanyl)propan-1-ol and 3-(methylsulfanyl)propanal were characterized by distinct roasted meat-like and musty tomato-like notes, respectively. These compounds were identified as key trace aroma components shaping the flavor profile of aged *Nongxiangxing* Baijiu. Notably, their roles in alcoholic beverages parallel findings in wine studies, where sulfur compounds (e.g., methionol) have been extensively linked to methionine degradation pathways during fermentation and aging [31,32].

The concentration of linalool, a monoterpenoid compound, was observed to initially increase with aging duration but exhibited a slight decline in longer-aged samples. Characterized by floral and citrus-like notes, linalool likely contributed to the enhanced fruity and floral flavor profile of aged Baijiu. This biphasic trend aligns with findings in wine chemistry: Slaghenaufi reported that linalool levels rise during early oxidative stages but eventually diminish, a pattern attributed to oxidative degradation or volatilization losses [33].

## 3. Material and Methods

### 3.1. Baijiu Samples

Baijiu samples were produced using the traditional manufacturing process by GuJingGong Co. Ltd. (Bozhou, China) and were aged under uniform storage conditions without blending. All test samples for this study were sourced from specific years (0, 3, 5, 11, and 15) and were bottled in glass containers. Prior to testing, the samples were kept at room temperature for a period of 5 days. Supplementary Table S2 provides a summary of the sample details.

### 3.2. Reagents and Chemicals

Sodium chloride (analytical reagent) and anhydrous sodium sulfate (analytical reagent) were sourced from China National Pharmaceutical Group Co. (Shanghai, China). Ethanol (HPLC grade) and dichloromethane (HPLC grade) were acquired from Sigma Aldrich (St. Louis, MO, USA). All chemicals and internal standards purchased had a purity of over 95% and were supplied by Sigma Aldrich (St. Louis, MO, USA) and J&K Scientific Co., Ltd. (Beijing, China). Deionized water was obtained from the Milli-Q purification system (Millipore, Bedford, MA, USA).

### 3.3. Sensory Analysis

According to Ma, et al. [34], sensory analysis was conducted using screened and trained panels. The panelists were recruited from Jiangnan University’s Laboratory of Brewing Microbiology and Applied Enzymology and received training on 54 odor standards to identify various odor attributes, guided by “Le nez du vin” by Jean Lenoir from Provence, France. After one year of training, the participants were able to distinguish odorants and assess aroma intensity. The final panel consisted of 20 panelists (10 male and 10 female), all of whom provided written consent. All samples were evaluated by the same panel of panelists.

Following the International Standard Method System for Sensory Analysis (ISO 8586:2012) [35], sensory profiles for various batches of Baijiu samples were established. Expert masters from the company selected representative Baijiu samples for analysis by a panel tasked with identifying specific descriptions and confirming the aroma intensity of each sample.

For this, 10 mL aliquots of each Baijiu sample were poured into a clear standard tasting cup labeled with a random three-digit code. To avoid fatigue, they had a 5-min rest between each tasting group. The panelists then graded the strength of each descriptor for each sample on a scale of 0 (very weak) to 12 (very strong). Every sample needs to be evaluated three times.

### 3.4. Baijiu Analysis

#### 3.4.1. Extraction of Volatile Compounds by Liquid–Liquid Extraction (LLE)

Volatiles were extracted by LLE using a previously described procedure [9]. Baijiu samples (50 mL) were diluted in 10 vol% ethanol with boiled ultrapure water, saturated with NaCl, and then extracted with dichloromethane (3 × 50 mL). The mixed extracts were dried with anhydrous Na_2_SO_4_ for 1 d and then concentrated under a gentle stream of nitrogen to a final volume of 500 μL. Prior to GC-O analysis, the concentrated fractions were stored at −20 °C.

#### 3.4.2. Aroma Extraction by Headspace Solid-Phase Microextraction Arrow (HS-SPME-Arrow)

The volatile compounds in Baijiu were extracted using HS-SPME-Arrow following the methodology outlined by Wang et al. [9]. The Baijiu samples were diluted to 10% alcohol by volume (ABV). Next, 5 mL of this solution was placed in a 20 mL headspace vial, with the addition of 1.5 g of sodium chloride (NaCl). The vial was then sealed with a PTFE/silicone septum and a screw top.

The incubation, extraction, and injection processes were performed using an automatic headspace sampling system (SPME-Arrow) from CTC Analytics AG, Zwingen, Switzerland. A 120 μm divinylbenzene/carbon wide range/polydimethylsiloxane (DVB/CAR WR/PDMS) fiber, also from CTC Analytics AG, was used to capture gas mixtures from the vial’s headspace. The sample was equilibrated at 50 °C for 5 min, followed by a 45-min extraction at the same temperature while stirring at a rotational speed of 300 rpm.

#### 3.4.3. GC-O Analysis

GC-O was performed using an Agilent 7890 gas chromatograph (Agilent Technologies, Santa Clara, CA, USA) connected to an olfactometry system (ODP 2, Gerstel, Mülheim an der Ruhr, Germany). DB-FFAP and DB-5 columns were employed for the GC-O analysis using the same experimental settings as for the GC–MS analysis. The sniffer port temperature was maintained at 250 °C during all tests. Four panelists from Jiangnan University’s Laboratory of Brewing Microbiology and Applied Enzymology participated in the GC-O analysis (two males and two females). The panelists had three years of training in characterizing odor attributes using 61 common aroma compounds and 54 fragrance standards (“Le nez du vin,” Jean Lenoir, Provence, France). Each panelist performed each analysis twice, and the odor was confirmed when it was identified four times.

#### 3.4.4. cAEDA Test

The contribution of each odorant identified by GC-O was examined using cAEDA to assess the differences in aroma compounds between young and aged Baijiu samples. cAEDA was performed using the DB-FFAP column. The aroma extracts were diluted with dichloromethane in a 1:1 ratio. Each panelist examined each diluted sample twice in succession. The highest dilution at which the aroma ingredient could be identified was defined as the FD factor. Retention indices (RIs), odor descriptors, and mass spectrometry (MS) data were compared with those of real standards to identify the aroma components (Std). Using n-alkane (C7–C30) standards, the RIs of each odorant on the DB-FFAP and DB-5 columns were determined.

### 3.5. Quantitative Analysis of Aroma Compounds

Depending on the properties and concentration ranges of the compounds, targeted and accurate quantitative procedures were established using various extraction methods combined with four instrumental analytical techniques, based on previous laboratory experience [9].

#### 3.5.1. Quantification by Gas Chromatography Using a Flame Ionization Detector (GC-FID)

GC-FID analysis using an Agilent 7890 gas chromatograph with a flame ionization detector was employed to quantify 21 compounds. Each Baijiu sample was spiked with three ISs (tertiary amyl alcohol, 2-ethylbutanoic acid (IS2), and amyl ethanoate (IS3) at final concentrations of 116.59, 107.7, and 131.78 mg/L, respectively. Then, 1 μL of the spiked sample was directly injected into the GC in the split mode (split ratio = 20:1). High-purity helium (99.999%) was used as the carrier gas at a flow rate of 1 mL/min. A CP-WAX 57CB column (50 m × 0.25 mm i.d., 0.20 μm film thickness, Agilent Technologies) was used for separation. The temperature of the column was set at 35 °C for 5 min, increased to 100 °C for 2 min at a rate of 4 °C/min, increased to 150 °C at a rate of 8 °C/min, and 200 °C at a rate of 15 °C/min and was then held at 200 °C for 25 min. The injector and detector temperature were both set at 250 °C. Standard solutions were prepared in aqueous ethanol (50 vol%) and were further diluted (1:1 ratio) to obtain a range of concentrations to construct the calibration curves.

#### 3.5.2. Gas Chromatography-Mass Spectrometry (GC–MS) Analysis

GC–MS analysis was performed using an Agilent 7890 gas chromatograph and an Agilent 5977B mass spectrometer (Agilent Technologies, Santa Clara, CA, USA). The samples were analyzed by using a DB-FFAP column (60 m × 0.25 mm i.d., 0.25 m film thickness, Agilent Technologies) and a DB-5 column (30 m × 0.25 mm i.d., 0.25 m film thickness, Agilent Technologies). The column carrier gas was high-quality helium (≥99.999%) at a flow rate of 2 mL/min. The oven temperature settings were 45 °C for 2 min, increasing to 230 °C at a 6 °C/min ratio, and 230 °C for 10 min (DB-FFAP) or 45 °C for 2 min, increase to 270 °C at a 6 °C/min ratio, and 270 °C for 10 min (DB-5). The Baijiu extracts (1 μL) were injected at 250 °C in the splitless mode with a 5 min solvent delay. The mass spectra were obtained in electron ionization mode using an ionization energy of 70 eV and a scan range of m/z 50–350 amu. The temperature of the ion source was 230 °C.

#### 3.5.3. Quantification by Liquid–Liquid Microextraction–Gas Chromatography-Mass Spectrometry (LLME-GC–MS)

Strongly polar compounds were quantified using LLME-GC–MS, following previously described methods. Each Baijiu sample (8 mL) was diluted in 10% ethanol (by volume) together with saturated NaCl solution and spiked with 40 μL of 4-(4-methoxyphenyl)-2-butanone (IS10, 100 mg/L) and 5 mL of dichloromethane. The sample was vortex mixed (500 rpm) for 5 min, and the organic phase was separated, dried overnight with anhydrous Na_2_SO_4_, concentrated to 200 μL under a gentle stream of nitrogen, and stored at −20 °C. The GC–MS conditions were the same as those described above.

### 3.6. Aroma Recombination and Omission Tests

Compounds with OAV ≥ 0.1 were mixed in a 60% ethanol solution to create a recombination model, which was then contrasted with the original olfactory characteristics of the 0-year-aged and 15-year-aged Baijiu samples. The real Baijiu samples and the simulated aroma model were subjected to sensory evaluation by a panel of 20 qualified individuals. Assessors rated the strength of each of the nine aroma descriptors on a scale from 0 to 12. To ascertain the significance of specific chemicals, omission tests were conducted using a triangle test, in accordance with ISO 4120:2021 [36].

The omission models were prepared by omitting each aroma component of the recombination model that had a strong positive relationship with aging time. Two recombination models and one omission model were presented simultaneously for evaluation. The 20 assessors were asked to select the sample with the most pronounced sensory difference from the other two, and the outcomes were confirmed by statistical analysis.

### 3.7. Statistical Analysis

The SPSS statistical software for Windows (version 26.0) was used to conduct statistical analyses (SPSS Inc., Chicago, IL, USA). PCA was mapped by XLSTAT 2019 analysis. Analysis of variance (ANOVA) was used to evaluate the quantitative analysis and aroma profiling data. Aroma compound interactions were analyzed using Sigma Plot 14.0.

## 4. Conclusions

The aging process of *Nongxiangxing* Baijiu was systematically investigated through integrated sensory and quantitative analyses, revealing the critical roles of trace aroma compounds in shaping its maturation-driven flavor profile. A total of 94 aroma compounds were identified via GC-O/MS, with 61 compounds selected for aroma recombination based on odor activity values (OAVs). Among these, 12 compounds exhibited significant correlations with aging duration, including 4-methyl-2-methoxyphenol, 3-(methylsulfanyl)propan-1-ol, 3-(methylsulfanyl)propanal, β-phenylethanol, benzaldehyde, and linalool, which were identified as key contributors to the smoky, roasted meat-like, musty tomato-like, floral, and sweet notes characteristic of aged Baijiu. Omission tests confirmed their indispensable sensory roles, with significant differences (*p* < 0.001) observed in reconstituted models lacking these compounds. This study provides actionable insights for the Baijiu industry to refine aging protocols and achieve consistent, high-quality flavor profiles.

## Figures and Tables

**Table 1:** The aroma compounds detected in Baijiu samples by cAEDA-GC-O.

No	Compound	RI ^a^	RIL ^b^	Odor Descriptor	FD ^c^			Identification ^d^
					15 years	5 years	0 years	
	Esters							
1	Ethyl ethanoate	893	902	Fruity	1024	1024	2048	RI, MS, Std, Aroma
2	Methyl hexanoate	1108	1183	Fruity	32	4	4	RI, MS, Aroma
3	Ethyl hexanoate	1183	1235	Fruity	2048	1024	1024	RI, MS, Std, Aroma
4	Pentyl butanoate	1199	1326	Banana	8	4	4	RI, MS, Aroma
5	Propyl hexanoate	1253	1293	Pineapple	64	16	16	RI, MS, Std, Aroma
6	Ethyl heptanoate	1271	1342	Pineapple	32	16	16	RI, MS, Std, Aroma
7	2-Methylpropyl hexanoate	1286	1362	Fruity	8	4	4	RI, MS, Std, Aroma
8	Butyl hexanoate	1352	1384	Fruity	8	4	4	RI, MS, Std, Aroma
9	Hexyl butanoate	1355	1388	Soapy, fruity	4	4	4	RI, MS, Aroma
10	Ethyl octanoate	1379	1434	Fruity, waxy	4	4	4	RI, MS, Std, Aroma
11	3-Methylbutyl butanoate	1402	1459	Banana	64	32	64	RI, MS, Std, Aroma
12	Ethyl nonanoate	1486	1533	Fruity	8	8	16	RI, MS, Std, Aroma
13	Hexyl hexanoate	1571	1609	Herbal, grass	4	4	8	RI, MS, Std, Aroma
14	Ethyl decanoate	1606	1634	Waxy, fruity	4	2	16	RI, MS, Std, Aroma
15	Diethyl butanedioate	1659	1677	Fruity	16	4	4	RI, MS, Std, Aroma
16	Ethyl dodecanoate	1803	1860	Waxy, soapy	4	8	8	RI, MS, Std, Aroma
17	Ethyl butanoate	1023	1025	Fruity	1024	1024	512	RI, MS, Std, Aroma
18	3-Methylbutyl ethanoate	1128	1131	Sweet, fruity	4	16	16	RI, MS, Std, Aroma
19	Ethyl pentanoate	1122	1159	Fruity	32	16	8	RI, MS, Std, Aroma
20	Ethyl hexadecanoate	2255	2265	Waxy, fruity	2	4	4	RI, MS, Std, Aroma
21	Ethyl (9*Z*)-octadec-9-enoate	2479	2476	Fatty, oily	2	2	2	RI, MS, Std, Aroma
22	Ethyl (9*Z*,12*Z*)-octadeca-9,12-dienoate	2521	2521	Fatty, oily	4	2	2	RI, MS, Std, Aroma
23	Ethyl 2-methyl butanoate	1040	1053	Fruity	256	64	32	RI, MS, Std, Aroma
24	Ethyl 3-methylbutanoate	1055	1060	Sweet, apple	128	64	64	RI, MS, Aroma
25	Ethyl 4-methylpentanoate	1189	1197	Fruity	64	32	16	RI, MS, Std, Aroma
26	Ethyl 2-hydroxy-4-methylpentanoate	1508	1516	Blackberry	4	8	16	RI, MS, Aroma
27	Ethyl cyclohexanecarboxylate	1414	1424	Fruity	32	64	32	RI, MS, Std, Aroma
	Alcohols							
28	Butan-2-ol	938	1020	Sweet	512	256	256	RI, MS, Aroma
29	Propan-1-ol	972	1035	Alcoholic	64	32	16	RI, MS, Std, Aroma
30	2-Methylpropan-1-ol	1049	1078	Winey	4	4	4	RI, MS, Aroma
31	Pentan-2-ol	1078	1114	Oily, fermented	256	128	256	RI, MS, Std, Aroma
32	Butan-1-ol	1092	1137	Fusel, sweet	128	64	16	RI, MS, Aroma
33	3-Methylbutan-1-ol	1135	1201	Fermented	128	64	64	RI, MS, Aroma
34	Hexan-2-ol	1190	1210	Winey	16	8	8	RI, MS, Aroma
35	3-Methylbutan-2-ol	1220	1256	Fermented, Fruity	4	4	4	RI, MS, Aroma
36	3-Methylbut-3-en-1-ol	1225	1246	Sweet, fruity	4	4	4	RI, MS, Aroma
37	Heptan-2-ol	1274	1306	Citrus, herbal	4	8	4	RI, MS, Std, Aroma
38	Hexan-1-ol	1313	1341	Herbal	64	32	4	RI, MS, Aroma
39	Octan-3-ol	1430	1462	Mushroom	32	32	64	RI, MS, Std, Aroma
40	Heptan-1-ol	1410	1442	Green, herbal	16	16	32	RI, MS, Std, Aroma
41	Octan-1-ol	1514	1554	Waxy	16	4	4	RI, MS, Std, Aroma
42	2-Methylbutan-1-ol	1204	1197	Fusel, alcoholic	8	8	8	RI, MS, Std, Aroma
43	(±)-Butane-2,3-diol	1594	1604	Fruity, creamy	4	4	16	RI, MS, Aroma
	Acids							
44	Ethanoic acid	1415	1424	Sour	32	32	32	RI, MS, Std, Aroma
45	2-Methylpropanoic acid	1532	1555	Sour	4	8	64	RI, MS, Aroma
46	Butanoic acid	1591	1602	Cheese	64	32	32	RI, MS, Std, Aroma
47	3-Methylbutanoic acid	1637	1655	Sour, cheesy	2	8	16	RI, MS, Aroma
48	Pentanoic acid	1708	1733	Sweaty, sour	2048	1024	256	RI, MS, Std, Aroma
49	4-Methylpentanoic acid	1773	1800	Pungent, Cheesy	8	4	4	RI, MS, Std, Aroma
50	Hexanoic acid	1818	1857	Sour, fatty	16	16	2	RI, MS, Std, Aroma
51	Heptanoic acid	1928	1960	Sour, cheesy	8	4	2	RI, MS, Std, Aroma
52	Octanoic acid	2035	2070	Sweaty, cheesy	4	4	2	RI, MS, Std, Aroma
53	Propanoic acid	1530	1535	Cheesy	16	8	2	RI, MS, Std, Aroma
54	Nonanoic acid	2162	2169	Waxy, cheesy	16	16	64	RI, MS, Std, Aroma
	Aldehydes and ketones							
55	Oct-1-en-3-one	1309	1306	Mushroom, herbal	16	32	64	RI, MS, Std, Aroma
56	Oct-3-en-2-one	1388	1408	Earthy, herbal	8	8	16	RI, MS, Aroma
57	1,1-Diethoxyethane	892	892	Green, nutty	512	64	64	RI, MS, Std, Aroma
58	3-Methylbutanal	928	952	Aldehydic, fatty	32	16	4	RI, MS, Std, Aroma
59	Pentan-2-one	1009	1003	Fruity	16	16	4	RI, MS, Std, Aroma
60	3-Hydroxybutan-2-one	1280	1305	Buttery, creamy	32	8	4	RI, MS, Std, Aroma
61	Butane-2,3-dione	1005	977	Buttery, sweet	8	8	4	RI, MS, Std, Aroma
62	(*E*)-Oct-2-enal	1430	1433	Green, cucumber	128	32	16	RI, MS, Std, Aroma
63	(*E*)-Non-2-enal	1542	1551	Cucumber, green	64	32	32	RI, MS, Std, Aroma
64	(*E*,*Z*)-Nona-2,6-dienal	1572	1591	Cucumber, green	32	8	4	RI, MS, Std, Aroma
65	(*E*,*E*)-Nona-2,4-dienal	1696	1686	Fatty, green	16	16	4	RI, MS, Std, Aroma
66	(*E*,*E*)-Deca-2,4-dienal	1819	1802	Citrus, green	64	64	4	RI, MS, Aroma
67	(*E*)-Hept-2-enal	1309	1314	Green, fatty	4	4	2	RI, MS, Std, Aroma
	Terpenoids							
68	β-damascenone	1818	1827	Floral	512	64	32	RI, MS, Std, Aroma
69	β-Ionone	1947	1958	Floral	64	32	32	RI, MS, Aroma
70	Linalool	1552	1579	Floral, citrus	32	4	2	RI, MS, Aroma
71	Nerol	1806	1821	Floral	16	4	4	RI, MS, Aroma
	Lactones							
72	Butan-4-olide	1611	1626	Creamy, oily	64	32	32	RI, MS, Std, Aroma
73	Hexan-4-olide	1695	1703	Herbal	4	2	2	RI, MS, Std, Aroma
74	Nonan-4-olide	2031	2024	Coconut	128	64	128	RI, MS, Std, Aroma
	Aromatics							
75	β-phenylethanol	1923	1906	Floral	256	128	16	RI, MS, Std, Aroma
76	2-Phenylethanal	1634	1642	Green, sweet	128	16	8	RI, MS, Std, Aroma
77	Benzaldehyde	1508	1534	Fruity	8	4	2	RI, MS, Std, Aroma
78	Ethyl 3-phenylpropionate	1870	1880	Floral, honey	2048	256	256	RI, MS, Std, Aroma
79	Ethyl 2-phenylethanoate	1764	1797	Floral	128	128	16	RI, MS, Std, Aroma
	Furans							
80	2-(Diethoxymethyl)furan	1442	1432	Mushroom	16	16	64	RI, Aroma
81	Furan-2-carbaldehyde	1458	1466	Baked bread	16	32	64	RI, MS, Std, Aroma
82	1-(Furan-2-yl)pentan-1-one	1737	1747	Sweet, caramel	2	4	64	RI, Aroma, Aroma
83	(Furan-2-yl)methanol	1666	1647	Bready	4	4	8	RI, MS, Std, Aroma
	Sulfur-containing compounds							
84	2-(Methylsulfanyl)ethan-1-ol	1515	1486	Sulfurous, meaty	256	32	32	RI, MS, Aroma
85	3-(Methylsulfanyl)propanal	1455	1458	Musty tomato	2048	128	16	RI, MS, Std, Aroma
86	3-(Methylsulfanyl)propan-1-ol	1719	1719	Meaty, onion	8	16	8	RI, MS, Std, Aroma
	Pyrazines							
87	2,3,5-Trimethylpyrazine	1397	1392	Nutty, earthy	32	4	4	RI, MS, Std, Aroma
88	2,3,5,6-Tetramethylpyrazine	1472	1477	Nutty	64	8	16	RI, MS, Std, Aroma
	Phenolic compounds							
89	4-Methyl-2-methoxyphenol	1945	1953	Woody	64	16	16	RI, MS, Std, Aroma
90	Phenol	1987	2030	Plastic, rubber	4	4	8	RI, MS, Std, Aroma
91	4-Ethyl-2-methoxyphenol	2022	2048	Smoky, clove	32	32	16	RI, MS, Std, Aroma
92	4-Methylphenol	2078	2089	Phenolic	256	128	128	RI, MS, Std, Aroma
93	4-Ethylphenol	2195	2195	Smoky	256	64	64	RI, MS, Std, Aroma
94	Vanillin	2599	2600	Vanilla	256	128	128	RI, MS, Std, Aroma

^a^ RI (retention index) is a standardized parameter in gas chromatography defining a compound’s retention behavior relative to n-alkane standards, calculated as a logarithmic function of retention times to enable consistent compound identification across different systems. ^b^ RIL (retention index library) is a comprehensive, curated database that stores experimentally determined or theoretically predicted retention index (RI) values of chemical compounds. ^c^ FD (flavor dilution factor), defined as the highest split ratio in the LLE fraction at which the odorant could be perceived by GC-O. ^d^ Identification based on RI (retention index), MS (mass spectrometry), Aroma (odor description by comparison to the reference standards by GC-O), and Std (standards).

**Table 2:** OAVs and their ratios of trace compounds in aged and young Baijiu.

No	Compound	Odor Threshold (μg/L)	OAV		G-15/G-0 Ratio ^j^
			G-15	G-0	
1	4-Methyl-2-methoxyphenol	314.56 ^b^	7.3	0.8	9.59
2	Heptan-1-ol	26,600 ^c^	0.1	<0.1	6.48
3	4-Ethyl-2-methoxyphenol	122.74 ^b^	16.8	3.6	4.67
4	Heptanoic acid	13,821.32 ^b^	2.6	0.6	4.44
5	β-damascenone	5.38 ^a^	3.0	0.8	3.95
6	Nonan-4-olide	90.66 ^b^	4.1	1.1	3.86
7	Octanoic acid	2701.23 ^b^	16.8	4.8	3.50
8	3-(Methylsulfanyl)propanal	7.1 ^b^	2.1	0.7	3.20
9	Hexan-4-olide	12500 ^e^	0.1	<0.1	2.89
10	2,3,5,6-Tetramethylpyrazine	80,073.16 ^b^	<0.1	<0.1	2.69
11	Vanillin	438.52 ^b^	0.1	<0.1	2.64
12	Diethyl butanedioate	353,193.25 ^b^	<0.1	<0.1	2.39
13	Pentanoic acid	389.11 ^b^	108.6	45.9	2.37
14	4-Ethylphenol	617.68 ^b^	2.1	0.9	2.35
15	Butan-2-ol	50,000 ^c^	1.8	0.8	2.15
16	2,3,5-Trimethylpyrazine	729.86 ^b^	0.6	0.3	2.12
17	Phenol	18,909.34 ^b^	<0.1	<0.1	2.02
18	Benzaldehyde	4203.1 ^b^	0.9	0.5	2.00
19	4-Methylphenol	166.97 ^b^	18.7	11.1	1.68
20	Propyl hexanoate	12,783.77 ^b^	0.7	0.4	1.64
21	Octan-1-ol	1100 ^c^	0.9	0.6	1.62
22	Nonanoic acid	3560 ^b^	0.4	0.3	1.51
23	2-Phenylethanal	262 ^b^	2.5	1.6	1.50
24	1,2-Propanediol	4660 ^f^	0.8	0.5	1.45
25	Ethyl heptanoate	13,153.17 ^b^	5.1	3.6	1.44
26	Ethyl 3-phenyl propionate	125.21 ^b^	79.1	55.4	1.43
27	β-phenylethanol	28,900 ^c^	0.2	0.1	1.41
28	3-(Methylsulfanyl)propan-1-ol	2110.41 ^b^	<0.1	<0.1	1.40
29	4-Methyl pentanoic acid	144 ^b^	43.0	31.5	1.37
30	Propanoic acid	18,200 ^b^	1.4	1.0	1.34
31	Ethyl 2-methylbutanoate	248.6 ^a^	0.7	0.5	1.30
32	(*E*)-Oct-2-enal	15.1 ^b^	22.9	18.5	1.24
33	Linalool	13.1 ^d^	1.3	1.1	1.22
34	Ethyl cyclohexanecarboxylate	21.37 ^a^	0.5	0.4	1.19
35	Furfuryl alcohol	54,700 ^c^	0.1	0.1	1.16
36	Pentan-2-ol	194,000 ^c^	0.1	0.1	1.16
37	Ethyl (9*Z*,12*Z*)-octadeca-9,12-dienoate	32,298.23 ^e^	0.6	0.6	1.10
38	2-methylpropyl hexanoate	5350.3 ^b^	14.1	13.7	1.03
39	3-Methylbutan-1-ol	37,400 ^c^	0.6	0.6	1.03
40	Ethyl palmitate	58,362.49 ^e^	0.7	0.7	0.97
41	Ethyl (9*Z*)-octadec-9-enoate	65,139.73 ^e^	0.1	0.2	0.94
42	Ethyl 2-phenylethanoate	406.83 ^b^	7.7	8.6	0.89
43	Butane-2,3-dione	100 ^c^	0.2	0.2	0.89
44	3-Methylbutyl butanoate	1400 ^b^	13.7	15.5	0.88
45	Ethyl 4-methylpentanoate	188.32 ^a^	0.6	0.7	0.84
46	Decanoic acid	2800 ^g^	1.1	1.4	0.81
47	3-Methylbutanoic acid	1045.47 ^b^	20.9	26.0	0.80
48	(±)-Butane-2,3-diol	50,000 ^h^	0.3	0.4	0.77
49	Butyl hexanoate	678 ^b^	17.0	23.9	0.71
50	2-Furaldehyde diethyl acetal	6172 ^i^	<0.1	<0.1	0.69
51	(*E*)-Hept-2-enal	2112.01 ^e^	0.1	0.1	0.66
52	Ethyl dodecanoate	640 ^c^	1.9	2.9	0.65
53	Ethyl decanoate	1122.3 ^b^	3.5	6.3	0.56
54	Hexyl hexanoate	1890 ^c^	5.8	11.0	0.53
55	3-Hydroxybutan-2-one	259 ^c^	5.7	11.8	0.48
56	2-Methylpropanoic acid	1580 ^b^	7.9	16.5	0.48
57	Furan-2-carbaldehyde	44,029.73 ^b^	0.1	0.2	0.45
58	(*E*)-Non-2-enal	50.5 ^b^	59.4	133.1	0.45
59	3-Methylbutyl ethanoate	93.93 ^b^	53.1	131.1	0.40
60	Heptan-2-ol	1433.94 ^b^	1.4	5.2	0.27
61	Octan-3-ol	6.12 ^b^	2.6	12.2	0.21
62	(*E*,*E*)-2,4-Decadienal	7.71 ^b^	7.7	38.5	0.20
63	Ethyl nonanoate	3150.61 ^b^	<0.1	0.6	0.04

^a^ Calculated in the laboratory; odor thresholds were calculated in a 60% water/ethanol solution. ^b^ Odor threshold previously reported in ref [10]; ^c^ Odor threshold previously reported in ref [15]; ^d^ Odor threshold previously reported in ref [16]; ^e^ Odor threshold previously reported in ref [17]; ^f^ Odor threshold previously reported in ref [18]; ^g^ Odor threshold previously reported in ref [19]; ^h^ Odor threshold previously reported in ref [20]; ^i^ Odor threshold previously reported in ref [21]; ^j^ G-15/G-0 Ratio is defined as the ratio obtained by dividing the OAV value of each compound G-15 by the OAV value of G-0.

**Table 3:** Omission test from complete reconstitution of aroma compounds.

No	Compounds	Odor Descriptor	N/20	Significance ^a^
1	Vanillin	Vanilla, sweet	3	-
2	4-Methyl-2-methoxyphenol	Smoky	18	***
3	4-Ethyl-2-methoxyphenol	Smoky	6	-
4	Benzaldehyde	Almond	11	**
5	β-phenylethanol	Rose, floral	13	**
6	3-(Methylsulfanyl)propan-1-ol	Roasted meat	16	***
7	Nonan-4-olide	Coconut	4	-
8	3-(Methylsulfanyl)propanal	Musty tomato	18	***
9	Linalool	Floral, citrus	11	**
10	2,3,5,6-Tetramethylpyrazine	Nut	1	-

^a^ Significance analysis of omission experiments. **, highly significant (*p* ≤ 0.01); ***, very highly significant (*p* ≤ 0.001), -, no significance (*p* > 0.05).

## Data Availability

The original contributions presented in this study are included in the article/supplementary material. Further inquiries can be directed to the corresponding author.

## Supplementary Materials

The following supporting information can be downloaded at: https://www.mdpi.com/article/10.3390/molecules30142963/s1, Supplementary Table S1. Calibration curves, linearity ranges, and recovery rates for 85 odor compounds. Supplementary Table S2. Information on the Baijiu samples.
