# Supplementary material for: Characterization of Key Aroma Compounds in Aged Chinese Nongxiangxing Baijiu Based on Sensory and Quantitative Analysis: Emphasis on the Contribution of Trace Compounds
Source: Molecules. 2025 Jul 14;30(14):2963. doi: 10.3390/molecules30142963 (PMC12298148; doi:10.3390/molecules30142963)
Supplement: Supplementary file 1 [file molecules-30-02963-s001.zip › molecules-3740838-supplementary.pdf]

Supplementary Table S1. Calibration curves, linearity ranges and recovery rates for 85 odor compounds

number	compound	quantitative method	quantitate ion	internal standard	slope	intercept	liner range (μg/L)	recovery %	R^2
1	ethyl hexanoate	GC-FID	-	amyl acetate	0.7184	0.3492	18.82~9635.47[a]	104.17	0.9996
2	hexanoic acid	GC-FID	-	2-ethylbutyric acid	1.1160	0.0198	0.92~470.05[a]	105.18	0.9980
3	ethyl 2-hydroxypropanoate	GC-FID	-	amyl acetate	1.2583	0.8767	18.82~9634.74[a]	96.88	0.9976
4	ethyl ethanoate	GC-FID	-	amyl acetate	1.4007	0.5663	17.44~8929.80[a]	111.52	0.9987
5	ethanoic acid	GC-FID	-	2-ethylbutyric acid	3.2965	0.9768	53.98~1727.43[a]	103.69	0.9886
6	propan-1-ol	GC-FID	-	tertiary amyl alcohol	1.4198	0.1878	8.32~4259.34[a]	108.12	0.9995
7	3-methylbutan-1-ol	GC-FID	-	tertiary amyl alcohol	0.8289	0.0195	1.68~430.67[a]	95.03	0.9990
8	ethyl butanoate	GC-FID	-	amyl acetate	0.5842	0.0020	0.69~88.22[a]	95.75	0.9985
9	ethanal	GC-FID	-	tertiary amyl alcohol	2.1561	-0.0125	1.36~697.58[a]	93.76	0.9986
10	butan-1-ol	GC-FID	-	tertiary amyl alcohol	0.9450	-0.0022	0.54~68.62[a]	97.04	0.9994
11	butanoic acid	GC-FID	-	2-ethylbutyric acid	0.6356	0.0366	1.25~159.83[a]	102.84	0.9930
12	2-methylpropan-1-ol	GC-FID	-	tertiary amyl alcohol	0.9130	0.0036	1.17~300.01[a]	98.71	0.9997
13	2-methylbutan-1-ol	GC-FID	-	tertiary amyl alcohol	0.8492	0.0037	1.75~224.23[a]	96.51	0.9994
14	1,1-diethoxyethane	GC-FID	-	tertiary amyl alcohol	1.1156	0.0016	1.98~506.68[a]	97.03	0.9995
15	1-hexanol	GC-FID	-	tertiary amyl	0.8210	-0.0004	1.08~34.48[a]	99.83	0.9996

				alcohol					
16	ethyl pentanoate	GC-FID	-	amyl acetate	0.5020	0.0014	0.58~36.80[a]	93.52	0.9979
17	methanol	GC-FID	-	tertiary amyl alcohol	4.6882	0.0558	2.99~191.68[a]	117.60	0.9372
18	ethyl octanoate	GC-FID	-	amyl acetate	0.3408	0.0001	0.19~6.07[a]	97.18	0.9989
19	butan-2-ol	GC-FID	-	tertiary amyl alcohol	1.0250	0.0030	0.97~30.98[a]	100.00	0.9997
20	2-methylpropyl hexanoate	LLE-GC-MS	99	hexanol-d13	1.6715	9.3782	78.88~19983.87	86.86	0.9926
21	ethyl heptanoate	GC-FID	-	amyl acetate	0.6102	0.0026	1.03~32.85[a]	97.77	0.9995
22	3-methylbutanal	GC-FID	-	tertiary amyl alcohol	0.5353	0.0081	0.84~53.67[a]	83.54	0.9865
23	octanoic acid	GC-FID	-	2-ethylbutyric acid	0.7352	0.0027	0.45~14.30[a]	109.07	0.9928
24	pentanoic acid	GC-FID	-	2-ethylbutyric acid	1.2002	0.0001	0.62~79.38[a]	103.09	0.9988
25	ethyl hexadecanoate	GC-FID	-	amyl acetate	0.4111	0.0063	0.57~36.70[a]	86.04	0.9906
26	heptanoic acid	GC-FID	-	2-ethylbutyric acid	1.0299	0.0007	0.44~14.15[a]	105.22	0.9944
27	propanoic acid	GC-FID	-	2-ethylbutyric acid	2.0528	0.0517	2.65~339.32[a]	100.85	0.9976
28	3-methylbutyric acid	GC-FID	-	2-ethylbutyric acid	0.9120	0.0054	0.59~75.43[a]	100.86	0.9996
29	pentan-1-ol	GC-FID	-	tertiary amyl alcohol	0.8652	-0.0010	0.83~53.03[a]	98.27	0.9997
30	ethyl (9Z,12Z)-octadeca-9,12-dienoate	GC-FID	-	amyl acetate	0.3700	0.0065	0.65~41.62[a]	82.74	0.9971
31	3-methylbutyl hexanoate	GC-FID	-	amyl acetate	0.7400	-0.1000	0.39~50[a]	87.49	0.9916
32	ethyl formate	GC-FID	-	amyl acetate	1.8214	0.0412	2.52~322.90[a]	92.12	0.9966
33	pentan-2-ol	GC-FID	-	tertiary amyl alcohol	0.9110	0.0007	0.83~53.17[a]	104.40	0.9987
34	diethyl butanedioate	SPME-GC-MS	101	phenethyl acetate-	18.2380	-1.4188	338.81	97.83	0.9918

				d3			~173473.16		
35	(±)-butane-2,3-diol	GC-FID	-	tertiary amyl alcohol	0.8093	0.0381	0.80~204.35[a]	87.61	0.9930
36	2-methyl-propanoic acid	GC-FID	-	2-ethylbutyric acid	1.2818	0.0089	1.27~81.18[a]	99.04	0.9994
37	butyl hexanoate	GC-FID	-	amyl acetate	0.6488	0.0009	0.42~13.35[a]	98.17	0.9996
38	hexyl hexanoate	SPME-GC-MS	117	phenethyl acetate-d3	1.1058	1.4839	62.78 ~32143.55	113.36	0.9909
39	ethyl 3-phenylpropionate	SPME-GC-MS	104	phenethyl acetate-d3	2.2718	0.1151	28.69 ~14686.99	111.25	0.9968
40	ethyl (9Z)-octadec-9-enoate	GC-FID	-	amyl acetate	0.4192	0.0051	0.57~36.58[a]	81.86	0.9964
41	propyl hexanoate	SPME-GC-MS	99	phenethyl acetate-d3	2.1533	0.5901	30.90 ~15821.51	117.70	0.9900
42	4-methyl pentanoic acid	SPME-GC-MS	57	pivalic acid	186.6600	0.1192	7.00 ~3584.49	116.61	0.9903
43	β-phenylethanol	GC-FID	-	tertiary amyl alcohol	0.5021	0.0047	0.41~52.03[a]	80.12	0.9986
44	3-methylbutyl ethanoate	SPME-GC-MS	70	phenethyl acetate-d3	4.7783	-0.4935	80.05~40986.67	117.68	0.9971
45	(furan-2-yl)methanol	SPME-GC-MS	98	hexanol-d13	45.9620	6.6046	120.40~61645.44	96.59	0.9957
46	furan-2-carbaldehyde	SPME-GC-MS	96	hexanol-d13	173.0000	0.1162	224.38 ~114882.72	114.37	0.9912
47	ethyl decanoate	SPME-GC-MS	88	phenethyl acetate-d3	8.0600	0.7100	7.81~2000	86.48	0.9945
48	benzaldehyde	GC-FID	-	tertiary amyl alcohol	0.6030	0.0064	0.47~15.03[a]	83.69	0.9829
49	decanoic acid	GC-FID	-	2-ethylbutyric acid	14.7800	-0.0129	0.52 ~ 26.49[a]	97.12	0.9889
50	4-methylphenol	SPME-GC-MS	107	guaiacol-d3	17.8530	0.0352	9.85 ~5042.17	80.41	0.9969

51	ethyl 2-phenylethanoate	GC-FID	-	amyl acetate	0.5466	0.0010	0.29~9.40[a]	83.21	0.9869
52	(E)-non-2-enal	SPME-GC-MS	55	hexanol-d13	5.6467	0.0408	15.41 ~7890.20	86.95	0.9976
53	2-methoxy-4-methylphenol	SPME-GC-MS	138	guaiacol-d3	11.1700	0.2672	3.88 ~3976.56	100.24	0.9972
54	4-ethyl-2-methoxyphenol	SPME-GC-MS	137	guaiacol-d3	4.3935	0.4729	5.56 ~2846.41	101.75	0.9942
55	heptan-2-ol	SPME-GC-MS	55	hexanol-d13	1.4436	0.0020	9.47 ~9700.40	112.28	0.9911
56	heptan-1-ol	SPME-GC-MS	70	hexanol-d13	0.5726	0.0784	5.93 ~3035.04	115.91	0.9975
57	3-hydroxybutan-2-one	GC-FID	-	tertiary amyl alcohol	1.2209	0.0299	2.23~284.85[a]	82.86	0.9986
58	4-ethylphenol	SPME-GC-MS	107	guaiacol-d3	6.2961	0.5574	11.47 ~5874.81	94.85	0.9983
59	ethyl dodecanoate	GC-FID	-	amyl acetate	0.5441	-0.0002	0.25~7.98[a]	95.37	0.9982
60	hexan-4-olide	LLE-GC-MS	85	guaiacol-d3	0.0083	0.0372	5.32~2722.23	103.76	0.9909
61	oct-1-en-3-one	LLE-GC-MS	70	octanal-d16	6.3952	0.0582	4.51~4635.35	92.50	0.9920
62	octan-1-ol	LLE-GC-MS	56	α-terpineol-d3	0.1146	-0.0485	27.75~14210.00	106.33	0.9998
63	pentan-2-one	LLE-GC-MS	86	octanal-d16	2.4232	0.0973	6.17~6315.68	91.75	0.9916
64	2-phenylethanal	SPME-GC-MS	120	acetophenone-d3	0.0035	0.0165	65.17~33368.33	101.36	0.9995
65	2,3,5-trimethylpyrazine	LLE-GC-MS	122	2-methylpyrazine-d6	0.1237	-0.0922	74.77~38281.64	103.58	0.9988
66	nonan-4-olide	LLE-GC-MS	85	furfural-d4	0.5190	0.01480	3.57~1829.46	98.74	0.9995
67	(E)-oct-2-enal	SPME-GC-MS	55	hexanol-d13	5.6381	0.019	1.34 ~687.30	85.85	0.9914
68	(2E,6Z)-nona-2,6-dienal	SPME-GC-MS	70	octanal-d16	0.0965	0.1251	16.04~8212.02	96.36	0.9955
69	2,3,5,6-tetramethylpyrazine	GC-FID	-	tertiary amyl alcohol	0.6965	0.0005	0.24-7.63a	97.76	0.9829
70	ethyl 2-methyl butanoate	LLE-GC-MS	102	2-methylpyrazine-d6	0.0114	-0.0474	382.66~195924.70	100.88	0.9999
71	(E)-hept-2-enal	SPME-GC-MS	55	hexanol-d13	0.2464	0.0143	0.65 ~332.82	115.79	0.9928
72	1,1-diethoxyethane	SPME-GC-MS	97	hexanol-d13	2.9692	0.0137	0.62 ~319.20	101.87	0.9957

73	(2E,4E)-nona-2,4-dienal	SPME-GC-MS	81	octanal-d16	0.2815	0.0342	1.57~802.68	93.36	0.8174
74	phenol	LLE-GC-MS	94	guaiacol-d3	0.1340	0.0029	16.38~8387.28	105.55	0.9999
75	ethyl 4-methylpentanoate	LLE-GC-MS	101	2-methylpyrazine-d6	0.0753	-0.0300	26.51~13574.49	98.12	0.9998
76	ethyl nonanoate	SPME-GC-MS	88	phenethyl acetate-d3	0.6923	0.0862	10.91 ~5586.46	115.26	0.9925
77	(2E,4E)-deca-2,4-dienal	SPME-GC-MS	81	hexanol-d13	0.2759	0.0284	0.70 ~356.68	103.22	0.9993
78	butane-2,3-dione	LLE-GC-MS	86	octanal-d16	6.2621	-0.5166	0.20~206.86	96.11	0.9962
79	linalool	LLE-GC-MS	93	α-terpineol-d3	0.0821	-0.0068	4.09~2093.46	110.33	0.9993
80	β-damascenone	LLE-GC-MS	69	hexanol-d13	0.0531	-0.0021	0.55~278.98	88.41	0.9989
81	oct-1-en-3-ol	SPME-GC-MS	57	hexanol-d13	0.1557	0.0006	1.68 ~861.36	88.64	0.9992
82	3-(methylsulfanyl)propanal	LLE-GC-MS	48	hexanol-d13	0.0033	-0.0020	4.87~28092.79	98.64	0.9989
83	ethyl cyclohexanoate	LLE-GC-MS	88	hexanol-d13	0.6740	-0.0083	0.57~147.04	112.98	0.9973
84	3-(methylsulfanyl)propan-1-ol	LLE-GC-MS	106	hexanol-d13	0.0006	-0.0208	2.93~750.04	99.75	0.9962
85	vanillin	LLE-GC-MS	166	guaiacol-d3	0.0719	-0.0066	1.72~882.00	96.35	0.9996

[a] represents the concentration range in units of mg/L.

Supplementary Table S2. Information of the baijiu samples

Sample name	Aging year	alcohol content (% vol)	Number of Batches
G-15	15	61	5
G-10	10	62	4
G-5	5	62	6
G-3	3	63	6
G-0	0	63	6
